# Comparative Analysis of Gut Microbial Composition and Functions in Przewalski’s Gazelle (*Procapra przewalskii*) From Various Habitats

**DOI:** 10.3389/fmicb.2022.913358

**Published:** 2022-06-08

**Authors:** Jingjie Zhang, Hongmei Gao, Feng Jiang, Daoxin Liu, Yuansheng Hou, Xiangwen Chi, Wen Qin, Pengfei Song, Zhenyuan Cai, Tongzuo Zhang

**Affiliations:** ^1^Key Laboratory of Adaptation and Evolution of Plateau Biota, Northwest Institute of Plateau Biology, Chinese Academy of Sciences, Xining, China; ^2^College of Life Sciences, University of Chinese Academy of Sciences, Beijing, China; ^3^Qinghai Provincial Key Laboratory of Animal Ecological Genomics, Xining, China; ^4^Qinghai Lake National Nature Reserve Bureau, Xining, China; ^5^Qinghai University, Xining, China; ^6^State Key Laboratory of Plateau Ecology and Agriculture, Qinghai University, Xining, China

**Keywords:** Przewalski’s gazelle, gut microbiome, environmental adaptability, 16S rRNA gene sequencing, pathogenic bacteria, co-occurrence network

## Abstract

Gut microbiota of mammals participates in host nutrient metabolism and plays an important role in host adaptation to the environment. Herein, to understand the relationship between environment differences and the composition and abundance of the gut microbiota of Przewalski’s gazelle (*Procapra przewalskii*) in almost all its habitats, high throughput sequencing of the 16S rRNA gene was used to compared the characteristics of the gut microbiota based on total 120 fecal samples. The results showed that Przewalski’s gazelle exhibited different characteristics of microbiota diversity in different habitats. The Jiangxigou Rescue Station (JX), Nongchang (NC), and Ganzihe and Haergai townships (GH) groups had a relatively high microbiota diversity, while the Niaodao scenic area (ND) group had the lowest diversity. This finding seemed to follow a similar pattern of change in the population of Przewalski’s gazelle. *Bacteroidetes* and *Actinobacteria* were the phyla with significant differences, especially between the Wayu township (WY) and the other groups. The difference in the microbiota mainly included the *Ruminococcaceae UCG-005*, *Christensenellaceae R-7 group*, and *Bacteroidaceae* and was enriched in the ND, WY, and other regions. We speculated that the difference in the gut microbiota was due to a difference in environmental characteristics, particularly the food resources that the host can obtain. We speculated that a similar microbiome has important functions for species survival and represents the evolutionary commonality of Przewalski’s gazelle, while a different microbiome plays an important role in the adaptation of Przewalski’s gazelle to a different environment. The results of this study illustrate how the same species adapts to different environments from the perspective of gut microbiota plasticity and therefore are of great significance for the protection and restoration of the population of this species.

## Introduction

There are many strategies for species to adapt to the environment, such as adjusting physiology and behavior and changing morphology to enable organisms to respond to changes in the living environment (Rosalino et al., 2014). Gut microbiota has strong plasticity and is considered to play an important regulatory role in the host’s environmental adaptability (Candela et al., 2010, 2012). The gut microbiota has evolved with the host and has formed a complex microecosystem with the animal gastrointestinal tract. It participates in important physiological activities such as food digestion, nutrient absorption, and immune regulation of the host and is crucial for the health of the host (Dethlefsen et al., 2007; Sampson and Mazmanian, 2015). In addition, the disturbance of the gut microbiota will also affect the health of the host.

The composition and function of gut microbiota are influenced by genetic background, age, different parts of the digestive tract, and external factors such as food composition, geographical environment, and season. Although the host genotype is considered important for shaping the gut microbiota, the external environment is also a crucial factor in controlling the gut microbiota and maintaining homeostasis in the intestine through interaction with the host (Spor et al., 2011). There is a great deal of evidence that environmental factors can change the bacteria and the community structure of the gut microbiota. The gut microbial diversity, functional gene diversity, metabolic pathway, and cellulolytic enzyme activity of captive giant pandas (*Ailuropoda melanoleuca*) were lower than those of wild pandas, while antibiotic resistance genes, heavy metal tolerance genes, and disease risk showed an increasing trend (Guo et al., 2019). Comparative studies of the gut microbiota of free-ranging and captive Namibian cheetahs (*Acinonyx jubatus*) showed no difference in microbial α diversity in captive individuals, but showed higher abundance of OTUs and transfer of disease-related functional pathways associated with potential pathogens (Wasimuddin et al., 2017). As Chinese Rhesus Macaques (*Macaca mulatta*) adapt to different geographical environments, new unique bacteria have been established to help macaques improve their adaptability under corresponding environmental conditions (Zhao et al., 2018).

Przewalski’s gazelle (*Procapra przewalskii*) is currently one of the most endangered ungulates in the world, which is also endemic to the Qinghai-Tibet Plateau. At present, almost all the individuals are believed to be distributed near the Qinghai Lake Basin in China. This species was once widely distributed in Western China. However, the population of this species has greatly declined and its habitat has shrunk due to habitat fragmentation and severe human disturbances. The current distribution range and activity area of this species are relatively small, and several independent small populations are scattered (Jiang et al., 2000; Li et al., 2012). The general environmental conditions of Przewalski’s gazelle are very similar. For instance, their habitat in the Qinghai-Tibet Plateau exhibits the characteristics of low temperature, low oxygen, and high radiation. There are certain differences of habitats for Przewalski’s gazelle. In particular, the vegetation types would directly lead to differences in the available food resources. It is commonly known that the diet of the majority of herbivores is compatible with plant diversity and composition found in their environment. Overwhelming evidence has shown that diet plays a key role in determining the composition of gut microbiota (Muegge et al., 2011; Scott et al., 2013; Flint et al., 2015; Graf et al., 2015). Therefore, we collected fecal samples of Przewalski’s gazelle from eight small geographical areas in the same season and performed the 16S rRNA gene sequencing. The chosen areas cover almost all the current ranges of the Przewalski’s gazelle. Our objectives are to (1) understand the gut microbial composition and the dominant bacteria in Przewalski’s gazelle in various regions, (2) analyze the differences in the gut microbiota of Przewalski’s gazelle between different regions as well as the possible reasons for the differences, and (3) explore the adaptation strategies of Przewalski’s gazelle to different environments from the perspective of gut microbiota.

## Materials and Methods

### Sample Collection

**Figure 1:**
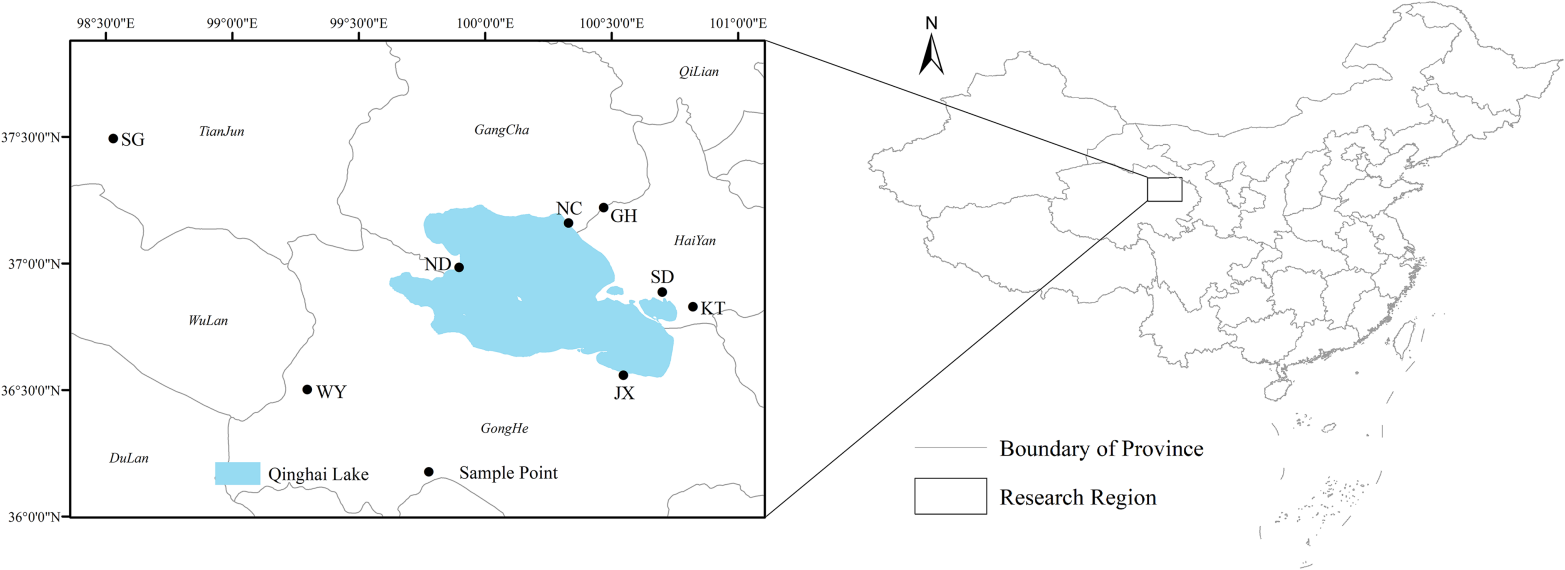
The eight geographical regions of the fecal samples of Przewalski’s gazelle.

Based on the ecological characteristics and current spatial distribution of the Przewalski’s gazelle, our previous results have divided the distribution of this species into 8 relatively independent habitat patches (Zhang et al., 2021). Therefore, during the winter of late 2018-early 2019, 15 fresh fecal samples of different Przewalski’s gazelle were collected from the Shengge township (SG), Wayu township (WY), Niaodao scenic area (ND), Ganzihe and Haergai townships (GH), Nongchang (NC), Shadao scenic area (SD), and Ketu township (KT), respectively, in Qinghai Province (Figure 1), covering almost all distribution areas of Przewalski’s gazelle with different vegetation types (Table 1). Additionally, we randomly collected 15 semi-captive fecal samples from the Jiangxigou Rescue Station (JX). Each fecal sample was taken using sterile disposable PE gloves and sterile sampling bags to avoid cross-contamination between the samples.

**Table 1:** Vegetation types in different study areas.

Study area	Vegetation formation and sub-formation	Vegetation type
Shengge township (SG)	Kobresia, Kobresia pygmaea, Stipa purpurea meadow, Stipa	Alpine grass and carex grassland, meadow of kobresia and miscellaneous grass
Jiangxigou Rescue Station (JX)	Stipa breviflora Griseb, Achnatherum splendens	Temperate tufted dwarf grass, dwarf semi-shrub desert steppe
Wayu township (WY)	Ceratoides	Typical grassland of temperate tufted grasses, desert of ceratoides, and desert of salt claw
Niaodao scenic area (ND)	Myricaria	Meadow of kobresia and miscellaneous grass, temperate deciduous thickets
Ganzihe and Haergai townships (GH)	Achnatherum, Artemisia, Blysmus sinocompressus, Carex spp. Meadow, Sabina, Spring barley, spring wheat, navew, rape field, Stipa	Typical grassland of temperate tufted grasses, temperate half shrub, low half shrub desert, meadow of kobresia and miscellaneous grass, typical grassland of temperate tufted grasses, one crop a year short growth period hardy crops
Nongchang (NC)	Spring barley, spring wheat, navew, rape field, Achnatherum	Typical grassland of temperate tufted grasses, one crop a year short growth period hardy crops
Shadao scenic area (SD)	Achnatherum	Typical grassland of temperate tufted grasses, temperate half shrub, low half shrub desert
Ketu township (KT)	Achnatherum, Artemisia, Kobresia	Temperate half shrub, low half shrub desert, typical grassland of temperate tufted grasses

All samples were temporarily preserved in liquid nitrogen and then stored at −80°C in an ultra-low temperature freezer in the laboratory. A total of 120 fecal samples were collected for DNA extraction, amplification, and sequencing. Furthermore, the vegetation types of Przewalski’s gazelle in different distribution areas were obtained by using 1:1 million vegetation map of China (Hou, 2019).^1^^,^^2^ There were distinct vegetation types in different distribution areas of Przewalski’s gazelle (Table 1).

### DNA Extraction, Amplification, and Sequencing

Genomic DNA from each fecal sample was extracted using an EZNA^®^ soil DNA kit (Omega Bio-tek, Norcross, GA, United States). The concentration and purity of DNA were then determined using a NanoDrop 2000 (Thermo Fisher Scientific, Waltham, MA, United States). Gel electrophoresis in a 1% agarose gel was used to assess the DNA quality. The V3-V4 hypervariable regions of the bacterial 16S rRNA gene were amplified by the microbial universal primers 338F (5′-ACTCCTACGGGAGGCAGCAG-3′) and 806R (5′-GGACTACHVGGGTWTCTAAT-3′).

PCR reactions were carried out in triplicate in a final volume of 20 μl consisting of 4 μl TransStart FastPfu buffer (5×), 2 μl dNTPs (2.5 mm), 0.8 μl upstream primer (5 μm) and 0.8 μl downstream primer (5 μm), 0.4 μl TransStart FastPfu DNA polymerase, 10 ng template DNA, and ddH_2_O to complete the 20 μl volume. Amplification reactions were performed in an ABI GeneAmp^®^ 9,700 PCR instrument (Applied Biosystems, Foster City, CA, United States) under the following conditions: 95°C for 3 min (initial denaturation); 27 cycles of denaturation at 95°C for 30 s, annealing at 55°C for 30 s, and extension at 72°C for 45 s; and 10 min at 72 C. Gel electrophoresis in a 2% agarose was used to evaluate PCR product yields. A genomic DNA library was constructed using the NEXTFLEX Rapid DNA-Seq Kit (Bioo Scientific, Austin, TX, United States). Sequencing was conducted on the Illumina Miseq PE300 platform (Illumina, San Diego, CA, United States). Sequences of approximately 410 bp were obtained.

### Genetic and Statistical Analyses

Quality control of the raw sequences was performed with Trimmomatic (version 0.39). The paired reads were merged into a sequence with a minimum overlap length of 10 bp using FLASH (version 1.2.7). The sequences were clustered into operational taxonomic units (OTUs) at 97% similarity using UPARSE (Edgar, 2013;^3^ version 7.1), and chimera were removed. The sequence with the highest frequency in each OTU was selected as the representative sequence for further annotation. Subsequently, to obtain the species classification information corresponding to each OTU, the Silva database (silva 132/16S) was adopted and the Ribosomal Database Project (RDP; version 2.11) classifier was used for taxonomic analysis of OTU representative sequences at 97% similar level. The confidence threshold was set to 0.8.

To estimate alpha diversity, the Shannon, sobs (the observed richness), ACE, and Chao1 indices were calculated using Qiime software at the OTU level (Caporaso et al., 2010). The principal coordinates analysis (PCoA) plot based on the Bray Curtis dissimilarity and unweighted and weighted UniFrac distance matrices were used to calculate the differences in beta diversity between each group, as well as to test the differences between the groups at the phylum and genus levels (Jiang et al., 2021). The community of the samples was clustered with a distance matrix and a hierarchical clustering tree of the samples was built. All the differences between the groups were analyzed using ANOSIM and Adonis. The potential pathogens were collected at phylum and genus level by literature search, and the differences between groups were analyzed.

A Wilcoxon signed-rank test and a Kruskal–Wallis rank-sum test was used with the STAMP software to test the species differences between the groups at a 95% confidence interval. FDR-based multiple test corrections were performed and Welch’s (uncorrected) 0.95 was used for post-hoc testing. To better understand the functions of the gut microbiota and their differences or similarities in various habitats of Przewalski’s gazelle, the Tax4Fun software package was used to transform the 16S taxonomic lineage based on the Silva database into the lineage of prokaryotes in the KEGG (Kyoto Encyclopedia of Genes and Genomes)^4^ and EggNOG databases. In addition, the correlation of the top 30 bacterial genera was calculated and the co-occurrence network of the bacteria was analyzed with Gephi v0.9.2 (Jacomy et al., 2014).

## Results

### 16S rRNA Sequencing Data

The total number of effective sequences obtained from 120 samples was 13,650,739, and the average length of the obtained sequences was 410 bp. According to the statistical results of the OTU species classification, 3,867 effective OTUs were extracted, screened and classified into 23 phyla, 43 classes, 110 orders, 208 families, and 473 genera.

A rarefaction curve was obtained by plotting the number of OTUs (*y*-axis) against the number of read (*x*-axis) per sample, showing that the curve gradually rises as the amount of sequencing increases (Supplementary Figure S1). When the amount of sequencing reached 48,100, the curve tended to be flat, indicating that the sequencing data were reliable and that the size of the samples from this sequencing was sufficient to reflect the diversity of the gut microbiota of Przewalski’s gazelle in the eight different areas.

### The Composition of the Gut Microbiota of Each Group

**Figure 2:**
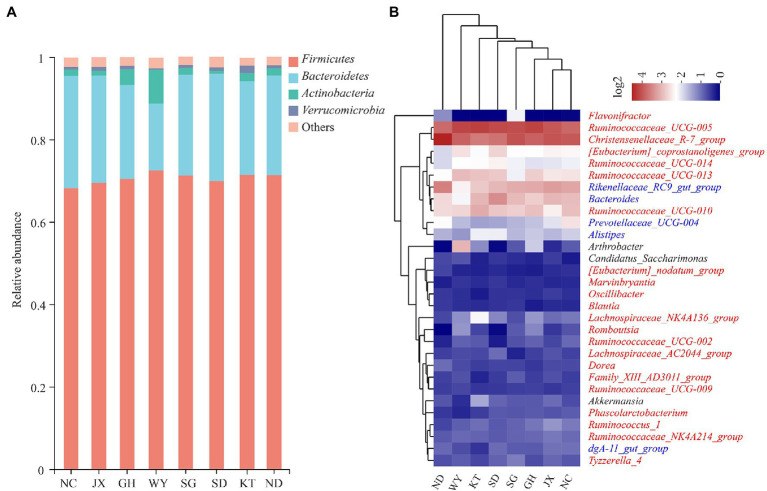
The composition of the gut microbiota of Przewalski’s gazelle. **(A)** Relative abundance of the dominant bacteria among the groups of Przewalski’s gazelle at the phylum level. **(B)** Cluster heat maps of the top 30 bacterial genera in terms of abundance.

The composition of the dominant phyla (relative abundance >1%) and the top 30 bacterial genera were analyzed. At the phylum level, *Firmicutes* and *Bacteroidetes* were the dominant phyla in each group (Figure 2A). Their cumulative relative abundance was higher than 88%. In the eight study locations JX, KT, NC, GH, SD, ND, SG, and WY, the relative abundances of *Firmicutes* were 69.46, 71.54, 68.25, 70.40, 69.89, 71.26, 71.20, and 72.61%, respectively, whereas the relative abundances of *Bacteroidetes* were 26.09, 22.71, 27.33, 22.89, 26.00, 24.21, 24.48, and 16.19%, respectively.

At the genus level, the dominant bacteria (relative abundance >1%) in Przewalski’s gazelle in all the study areas were *Ruminococcaceae UCG-005*, *Christensenellaceae R-7 group*, *Rikenellaceae RC9 gut group*, and *Bacteroides* (Figure 2B). The cluster heat map analysis showed that the gut microbiota of Przewalski’s gazelle in the ND and WY groups were far from the other six groups based on the top 30 bacterial genera. A relative abundance bar chart was plotted to visually show the distribution of the top four phyla and genera in each sample (Figure 2). Species composition and abundance are also illustrated by the graph.

### Analysis of Alpha and Beta Diversity

**Figure 3:**
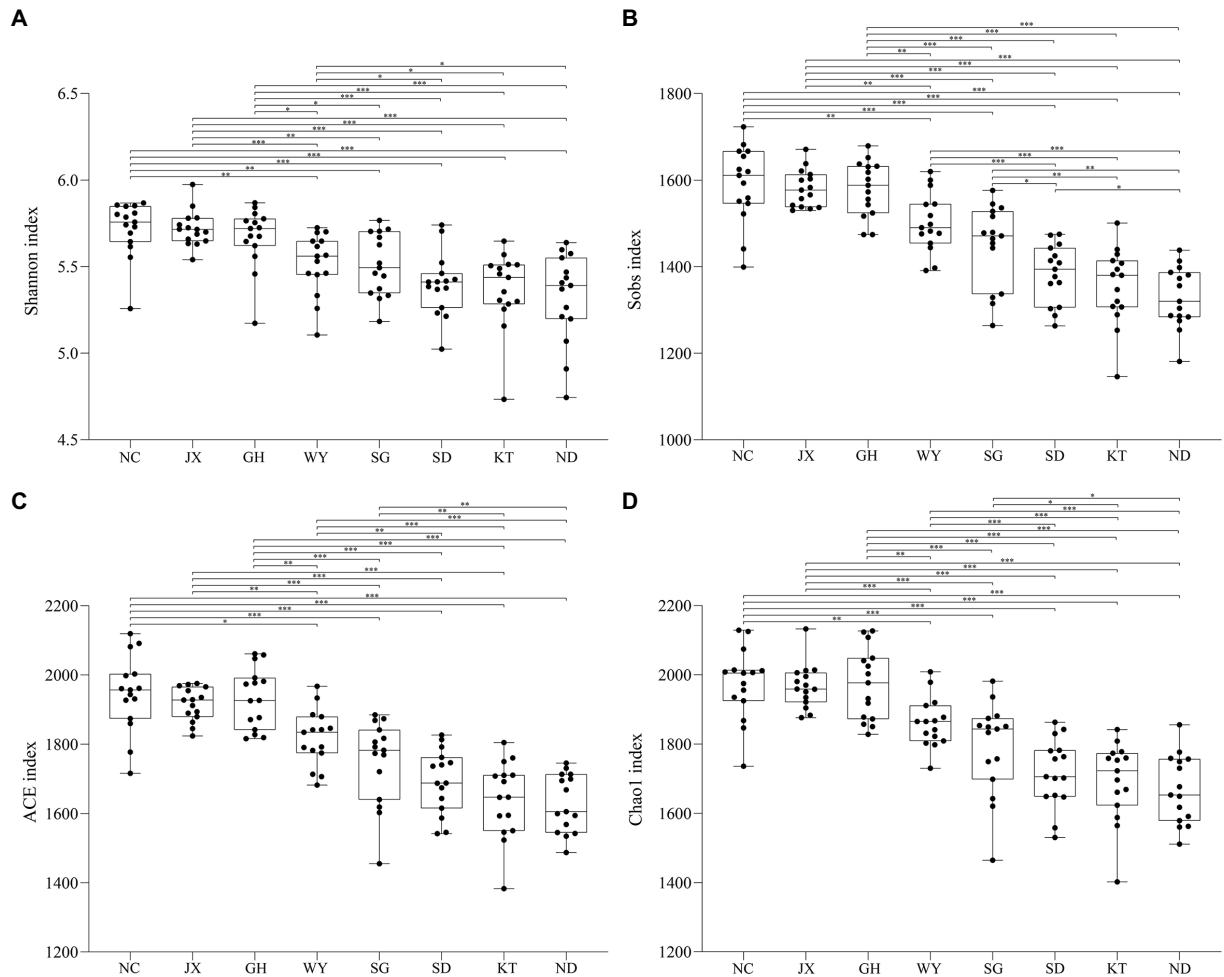
Alpha diversity indices of the gut microbiota of Przewalski’s gazelle found in the eight study areas. ^*^p < 0.05; ^**^p < 0.01; and ^***^p < 0.001.

The alpha diversity of the eight different groups showed that at the OTU level, the values of the Shannon and ACE indices in the JX, NC, and GH groups were higher than those of the other five groups, suggesting that the gut microbial richness and uniformity in the above-mentioned three groups were higher than in the other groups (Figures 3A**–**D). Nevertheless, the values of the Shannon and ACE indices in the ND group were the lowest (Figure 3).

**Figure 4:**
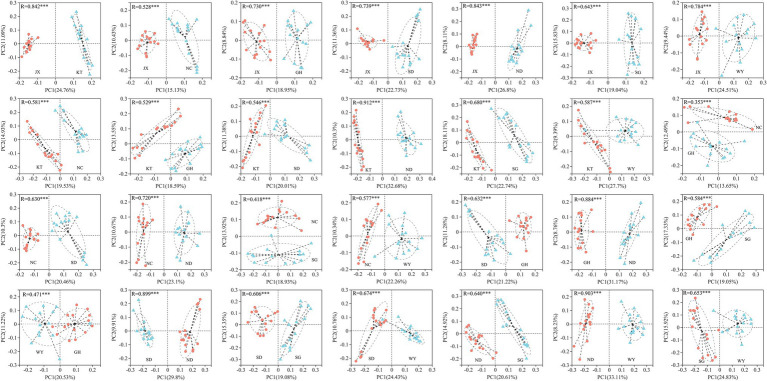
PCoA analysis of gut microbiome in Przewalski’s gazelle based on Bray-Curtis distance matrices. ^***^p < 0.001.

Beta diversity analysis was used to compare the differences in the gut microbiota of Przewalski’s gazelle in the different study areas based on three distance matrices (Bray Curtis, unweighted UniFrac, and weighted UniFrac) with ANOSIM and Adonis (Figure 4; Supplementary Table S1). The PCoA analysis showed that the differences in the gut microbiota of Przewalski’s gazelle between the eight groups were significantly greater than the differences within the groups at the OTU level.

**Figure 5:**
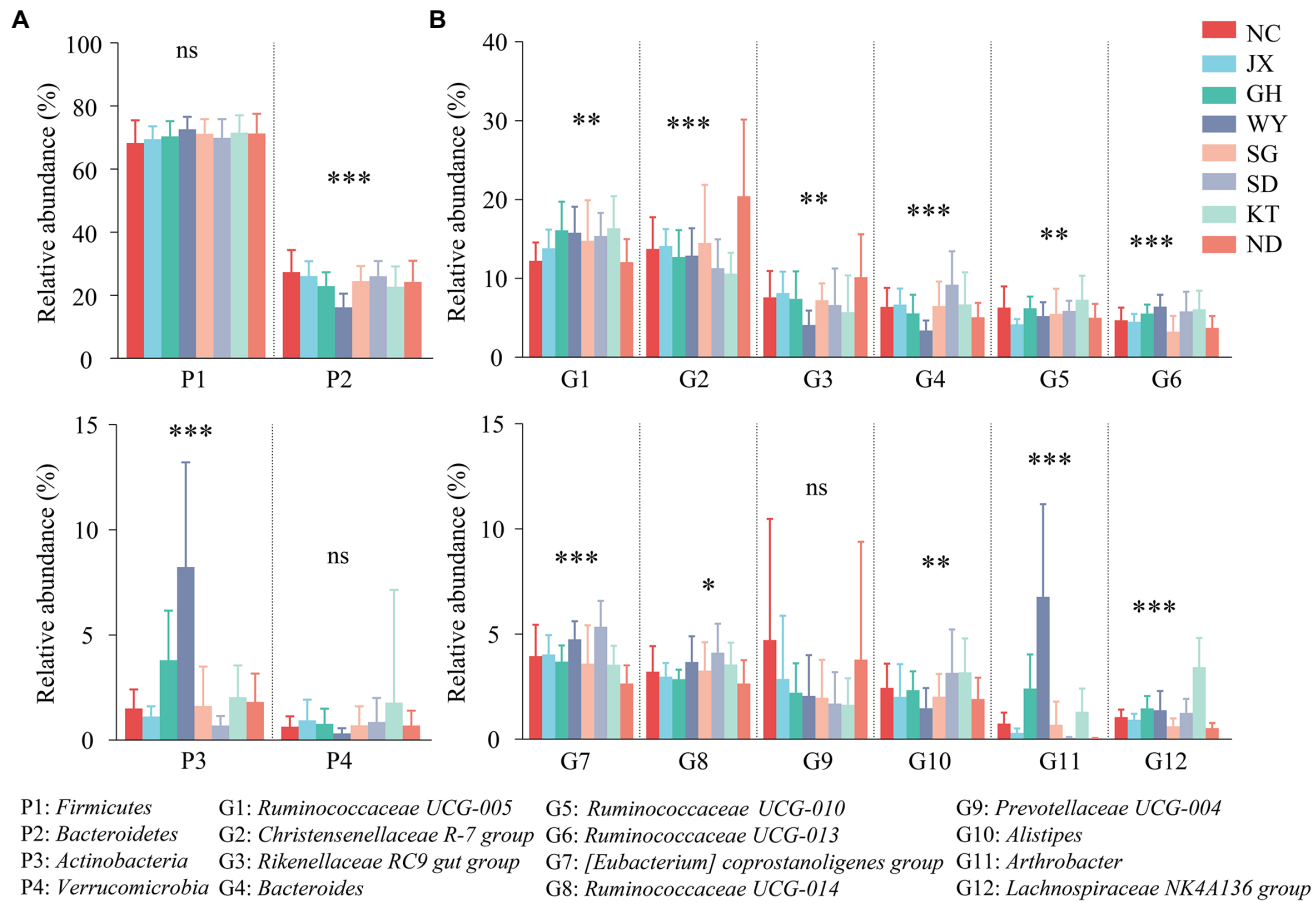
Analysis of the differences between the dominant bacteria in Przewalski’s gazelle at the phylum **(A)** and the genus **(B)** levels. ^*^p < 0.05; ^**^p < 0.01; and ^***^p < 0.001. ns, no significance.

The relative abundances of *Firmicutes* and *Verrucomicrobia* in the gut microbiota of Przewalski’s gazelle from all the regions showed no significant intergroup differences, while that of *Bacteroides* and *Actinobacteria* displayed significant intergroup differences (Figure 5A). Almost all the dominant genera in Przewalski’s gazelle from the different regions presented significant intergroup differences except for *Prevotellaceae UCG-004* (Figure 5B).

### Functional Prediction of the Gut Microbiota

**Figure 6:**
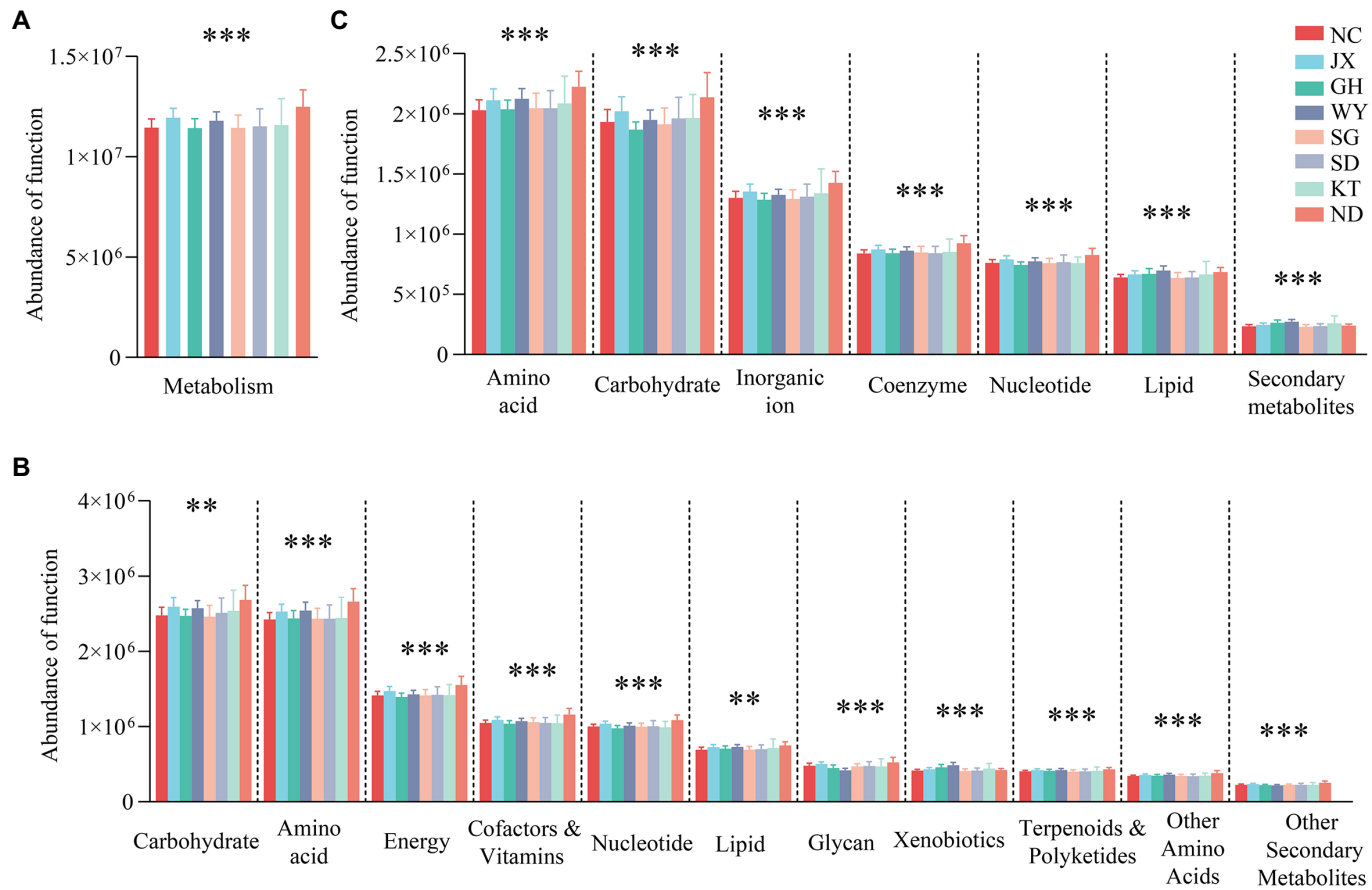
Analysis of the differences in metabolic functions between the groups based on the KEGG database at level 1 **(A)**, level 2 **(B)** and EggNOG database **(C)**. ^**^p < 0.01; and ^***^p < 0.001.

Based on the KEGG database, the gene function annotation analysis of the gut microbiome showed that the most important functions at level 1 were metabolism with a relative abundance (RA) value of 60.51% ± 0.2872, followed by environmental information processing (RA, 18.61% ± 0.2151) and genetic information processing (RA, 13.27% ± 0.0807). The functions exhibited significant differences, particularly the metabolic function of various substances of the gut microbiome among the eight groups at level 1 and level 2 (Figure 6). Moreover, the eight groups had different advantage functions. For example, the ND group had a higher abundance in the functions of organismal systems (i.e., environmental adaptation, digestive system, and immune system) and cellular processes (i.e., cell motility and eukaryotic community). The gut microbiota in the WY group in particular was significantly different from the other regions in the function of metabolism and organismal systems. For instance, metabolism of cofactors and vitamins, metabolism of terpenoids and polyketides, and biodegradation of xenobiotics had the highest abundance (*p* < 0.05), while glycan biosynthesis and metabolism, digestive system (*p* < 0.05), and immune system showed the opposite result (*p* < 0.05; Supplementary Table S2).

Based on the EggNOG database, the metabolic functions were selected for analysis. The results showed that amino acid transport and metabolism (8.34% ± 0.0729), carbohydrate transport and metabolism (7.86% ± 0.0829), and inorganic ion transport and metabolism (5.31% ± 0.0547) were the dominant metabolic functions of the gut microbiome in Przewalski’s gazelle. Analysis of the differences between the groups showed that all the metabolic functions of various substances differed significantly based on the KEGG and EggNOG databases (Figure 6).

### Analysis of Enterotypes and Pathogenic Bacteria

**Figure 7:**
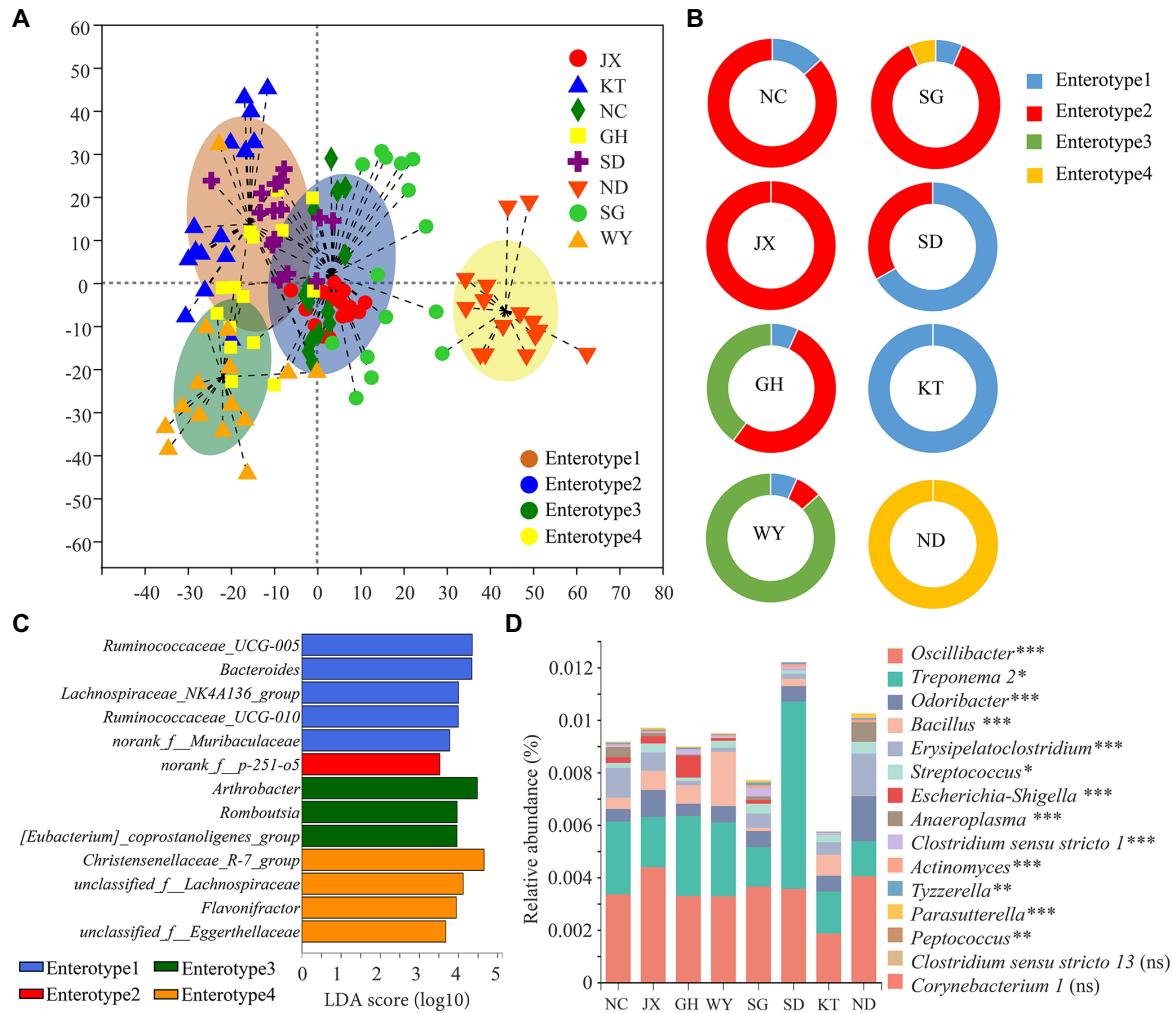
Analysis of the enterotype and the pathogenic bacteria of Przewalski’s gazelle from the different study areas. **(A)** Analysis of the diagram of the enterotype. **(B)** Analysis of the enterotype distribution of Przewalski’s gazelle in the different study areas. **(C)** Marker bacteria of each enterotype. **(D)** Analysis of the differences between the groups of the potentially pathogenic bacteria. ^*^p < 0.05; ^**^p < 0.01; and ^***^p < 0.001. ns, no significance.

The Jensen–Shannon divergence (JSD) distance matrices was selected to calculate the distance between the fecal samples of Przewalski’s gazelle in the different study areas based on the OTU relative abundance matrix. The larger the Calinski–Harabasz (CH) index, the closer the samples are within each cluster. In addition, the more dispersed the types, the better are the clustering results. Analysis of the enterotype diagram showed that the optimal number of clusters was 4 (Figure 7A). Consequently, the fecal samples of Przewalski’s gazelle from the eight areas could be divided into four enterotypes. In this study, 30, 55, 19, and 16 of the 120 samples of Przewalski’s gazelle belonged to enterotype 1, enterotype 2, enterotype 3, and enterotype 4, accounting for 25, 45.8, 15.8, and 13.3%, respectively. The SD and KT groups were mainly of enterotype 1, whereas the NC, SG, JX, and GH groups were mainly of enterotype 2, the WY group was mainly of enterotype 3, and the ND was mainly of enterotype 4 (Figure 7B). Marker bacteria of each enterotype were *Ruminococcaceae UCG-005*, *norank f p-251-o5*, *Arthrobacter,* and *Christensenellaceae R-7 group*, respectively (Figure 7C).

Furthermore, a total of 15 potentially pathogenic bacterial genera were obtained, 13 of which showed significant differences between the groups. We noted that *Oscillibacter* and *Treponema* 2 were the dominant potentially pathogenic bacteria. The SD group had the highest cumulative relative abundance of potential pathogens, while the KT group had the lowest cumulative relative abundance of potential pathogens (Figure 7D).

### Co-occurrence Network of the Core Bacteria

The co-occurrence network of the 30 most abundant genera revealed the relationships among the gut bacteria in the eight distribution areas of Przewalski’s gazelle. The results showed that the complexity of the gut microbial network structure of Przewalski’s gazelle was significantly different in the different distribution areas. The bacterial group in the SG group was the most complex since the bacteria in this group had more links (65 links), followed by the NC group (47 links) and the KT group (47 links).

**Figure 8:**
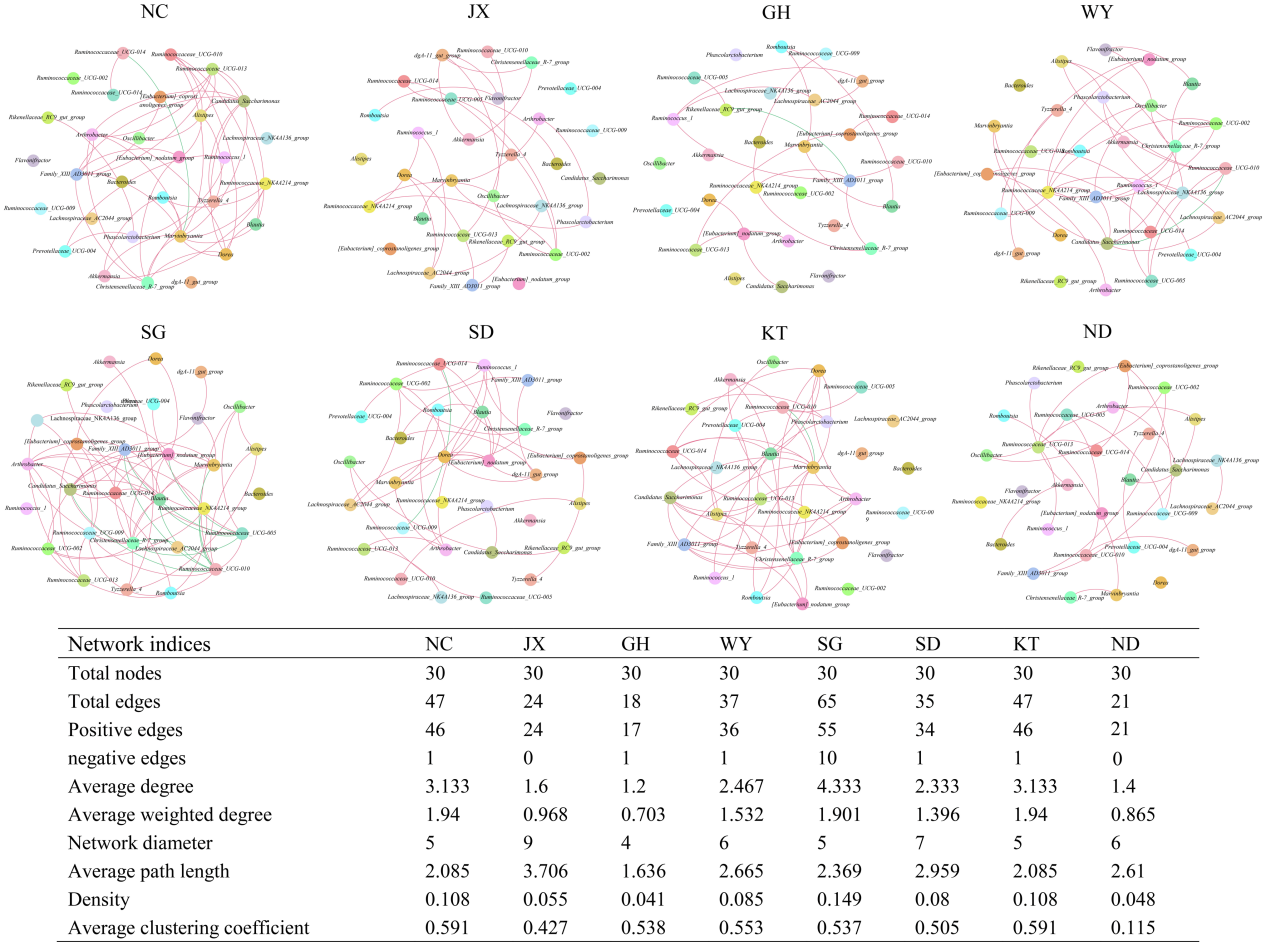
Co-occurrence networks of the 30 most abundant genera in Przewalski’s gazelle from eight different areas. Each line represents Pearson correlation coefficient, whose absolute value was higher than 0.5 and *p* value was less than 0.05. The red and green lines represent positive and negative correlations, respectively.

The bacterial genera with the greatest number of edges were *Ruminococcaceae UCG-010*, *Christensenellaceae R-7 group*, *Marvinbryantia*, *Ruminococcaceae UCG-005*, *Family XIII AD3011 group*, *Ruminococcaceae NK4A214 group,* and *Ruminococcaceae UCG-010* (Figure 8).

## Discussion

With the rapid development of sequencing technology, studies on gut microbial composition and diversity based on high-throughput sequencing technology can provide novel strategies for the protection of endangered wildlife. Due to the difficulty in collecting wildlife samples, there are very few studies on the gut microbiota of Przewalski’s gazelle. In this study, we applied high-throughput sequencing technology of the V3-V4 hypervariable regions of 16S rRNA to investigate the composition of the gut microbiota of Przewalski’s gazelle in its different distribution areas, as well as to establish a theoretical basis for implementing conservation measures of Przewalski’s gazelle backed by scientific research.

The study showed that Przewalski’s gazelle had similar dominant bacteria in all its distribution areas. This result indicated the presence of a commonality in the gut microbiota of Przewalski’s gazelle in different areas, which was also an essential outcome demonstrating the co-evolution of mammalian gut microbiota and their host. At the phylum level, both *Firmicutes* and *Bacteroidetes* were the dominant bacterial phyla, with a total proportion of over 88%, which is consistent with the conclusions of most studies (Samsudin et al., 2011; Ishaq and Wright, 2014; Li et al., 2017). The phylum *Firmicutes* can degrade fiber and cellulose into volatile fatty acids (Thoetkiattikul et al., 2013; Hu et al., 2017), and the phylum *Bacteroidetes* contributes to the breakdown of carbohydrates and proteins and improve nutrient utilization in the host (Waite and Taylor, 2014; Nuriel-Ohayon et al., 2016). In addition, both of them play an important role in enhancing host immunity and maintaining the balance of gut microbiota (Sears, 2005; Shang et al., 2016). The phylum *Actinobacteria* is also an essential component in each group. The members of this phylum play important roles in the degradation of cellulose and lignin (Heurich et al., 2015; Lewin et al., 2016). At the genus level, the co-occurrence network analysis showed that most of the bacteria at the core of the network belong to the family *Ruminococcaceae* in the rumen, which is closely related to food digestion. The genera *Ruminococcaceae UCG-005* and *Christensenellaceae R-7 group* were the most abundant genera. *Ruminococcaceae UCG-005* belongs to the family *Ruminococcaceae*, which are important bacteria involved in the degradation of cellulose and hemicellulose in the rumen (Matulova et al., 2008; La Reau et al., 2016). The bacteria can produce a large amount of cellulase and hemicellulase and convert dietary fiber in foods into various nutrients required by the host (La Reau and Suen, 2018), which plays a key role in food digestion and carbohydrate metabolism of ruminants. *Christensenellaceae R-7 group* is mainly involved in host amino acid and lipid metabolism (Waters and Ley, 2019). Moreover, these bacteria were considered to be potentially beneficial since they are involved in the positive regulation of the intestinal environment and are associated with immune regulation and healthy homeostasis (Kong et al., 2016; Kashtanova et al., 2020; Li et al., 2020). Dominant gut microbes are considered as the result of strong selection and long-term co-evolution with the host (Groussin et al., 2020). Furthermore, they play an effective role in ensuring the health of the species by coping with long-term environmental changes (Asnicar et al., 2017; Sun et al., 2019a). We speculated that these dominant bacteria can help Przewalski’s gazelle decompose cellulose, obtain nutrients and improve immunity more effectively, and adapt better to the various environment.

Diversity results revealed that the diversity and uniformity of the gut microbiota in Przewalski’s gazelle in the JX, GH, and NC regions were higher than that in other regions. In addition to feeding on local vegetation, Przewalski’s gazelle may receive additional supplementary feeding in winter in these three regions, such as oats. The GH area is currently the largest distribution area of Przewalski’s gazelle. The vegetation types and food resources in this area are relatively diverse, which are probably the main reason for the high microbial diversity (Tinker and Ottesen, 2016; Chi et al., 2019). The NC area has also sufficient food resources. The JX area belonged to a semi-captive environment where Przewalski’s gazelle can obtain the most food resources, as there is no competition for resources from domestic animals. In our opinion, a better living environment will increase the diversity of the gut microbiome of Przewalski’s gazelle, while the α diversity of gut microbiota in Przewalski’s gazelle in the ND area was the lowest compared to the other regions. We speculated that the limitations of these environmental factors would affect the species’ gut microbial diversity because the ND area is currently the smallest distribution area of Przewalski’s gazelle, with a single vegetation type and limited food resources. Studies have shown that a high diversity of gut microbiota is beneficial to the host’s health and is also a sign of maturity of the gut microbiome (Turnbaugh et al., 2007; Le Chatelier et al., 2013). We also found that the wild population of Przewalski’s gazelle is relatively higher in the GH and NC areas; therefore, we speculated that there is a certain connection between the diversity of the gut microbiome and the population number.

Przewalski’s gazelle is currently a species that occurs only in a narrow range around Qinghai Lake, with extremely small distribution area. However, there are still certain differences between the habitats of several independent small populations, which are mainly represented in the vegetation types. Vegetation in different regions determines in part the type of food available to Przewalski’s gazelle. The study revealed that in different habitats, the composition of gut microbiota in Przewalski’s gazelle was also different. This variation is most likely due to the species’ adaptation to diverse habitats. The relative abundance of *Bacteroidetes* was the lowest in the WY, while that of the *Actinobacteria* was the highest in the WY, and these values are significantly different from those found in the other regions. We noticed that there was an obvious difference between the vegetation types in the WY and the other areas. The habitat type of Przewalski’s gazelle in the WY area included *Ceratoides arborescens* and salt claw desert, a vegetation type in the area that are halophytes with high fiber content, but low carbohydrate and protein content. We speculated that the difference in abundance may be used to improve the adaptability of these populations in special environments (Gao et al., 2020). *Proteobacteria* were most abundant in the SG group, followed by the GH, WY, and JX groups, but less abundant in the SD, KT, and ND groups. Previous studies have indicated that the surge of *Proteobacteria* in the gut was a manifestation of the imbalance and instability of the gut microbial community structure (Shin et al., 2015). As a result, we speculated that gut microbiota experiences minor variations in a single habitat.

*Fibrobacteres* are an important phylum of cellulose-degrading bacteria (Ransom-Jones et al., 2012, 2014), yet they were not present in the SG and ND groups, which was a significant result. The absence of these bacteria in these areas may be related to the environment, probably because these areas lack specific plants that the bacteria require to degrade, or another bacterial structure evolved in the intestine and effectively replaced the function of the bacteria to decompose and utilize certain cellulose. *Rikenellaceae RC9* had the lowest abundance in the WY group, but the highest abundance in the ND group. There is evidence that *Rikenellaceae RC9* can not only play a potential role in the degradation of plant-derived polysaccharides but also have a function in lipid metabolism (He et al., 2015; Sun et al., 2019b; Tavella et al., 2021). Because Przewalski’s gazelle confronts more survival obstacles in the NC than in the other locations, a larger abundance of *Rikenellaceae RC9* may assist Przewalski’s gazelle to receive more nutrients from limited food. The diet of the species in the WY group, on the other hand, was predominantly halophyte, with more cellulose but less sugar or lipids, which may explain the low abundance of this bacteria in the species of this region.

The functional prediction analysis revealed that Przewalski’s gazelle in the WY area and the other regions exhibited unique characteristics in a variety of functions, including glycan biosynthesis and metabolism, metabolism of secondary metabolites, and xenobiotics biodegradation and metabolism. It is abundant in the aforementioned functions and has generated major differences in the other regions. In addition, Przewalski’s gazelle presented in the WY obvious low abundance and differences in organismal systems, such as environmental adaptation, digestive system, and immune system, confirming the particularity of Przewalski’s gazelle in the WY’s food resources. We speculated that these functional changes play an important role in the adaptation of Przewalski’s gazelle to a special environment. We intend to conduct a metagenomic study in the future to better understand the role and adaptability of the gut microbiota of Przewalski’s gazelle. We found that the enterotype and the geographic distance of Przewalski’s gazelle were related, with the SD and KT being very close and having a similar habitat type. The WY and ND have relatively independent geographical locations, which may be the main reason of the significant difference of the enterotypes from the other areas. The four enterotypes had different marker bacteria, and their relative abundance may be closely related to food composition. We also speculated that the difference in wild animal habitats is responsible for the composition and function of their gut microbiota.

*Bacteroides* have been shown to have a beneficial impact on the mucosal immune system (Malmuthuge et al., 2015) and have been proven to have an important influence on the development of immunological tolerance to commensal microbiota (Mazmanian et al., 2008). Nonetheless, *Bacteroides* are pathogenic bacteria that can cause endogenous infections when the immune system or gut microbiota malfunctions (Patrick, 2002; Wexler, 2007). *Oscillibacter* and *Treponema* 2 were found to account for a high proportion of potentially pathogenic bacteria. *Oscillatoria* is an anaerobic pathogenic bacterial genus that may induce intestinal metabolic dysfunction and metabolic diseases in the host (Hae-Jin et al., 2015). *Treponema* 2 can cause inflammation of the colon and is related to dysentery (Kelly et al., 2016). Research on the composition and abundance of the potentially pathogenic bacteria in Przewalski’s gazelle will help understand the physiology and the pathological conditions of the population in different environments, which is crucial for population recovery.

## Conclusion

In conclusion, this study compared the gut microbiota of almost all distribution areas of Przewalski’s gazelle. The results revealed that environmental differences affect the gut microbiota of the species. The dominant gut microflora of Przewalski’s gazelle distributed in various geographical areas had great similarities. These bacteria are likely to play an essential role in the long-term evolution of the host and gut microbiota. However, the gut microbiome of Przewalski’s gazelle in multiple regions differed also in species, abundance, and function. These differences are a result of adapting to different environments.

## Data Availability Statement

The data presented in the study are deposited in the NCBI GenBank, accession number PRJNA722780 (https://dataview.ncbi.nlm.nih.gov/object/PRJNA722780?reviewer=65b4tumbfel4l7nv7r96tlpoqa).

## Author Contributions

JZ and TZ conceived and designed the experiments. JZ wrote the first draft of the manuscript. JZ, HG, FJ, DL, YH, XC, WQ, PS, and ZC contributed to sampling and laboratory work. JZ, FJ, and HG conducted data analysis. All authors contributed to the article and approved the submitted version.

## Funding

This work was supported by Second Tibetan Plateau Scientific Expedition and Research Program (STEP; grant number 2019QZKK0501), Qinghai Key R&D and Transformation Program (grant number 2019-SF-150), Qinghai Province Science and Technology Plan (grant number 2021-ZJ-951Q), and the Joint Grant from the Chinese Academy of Sciences-People’s Government of Qinghai Province on Sanjiangyuan National Park (LHZX-2020-01).

## Conflict of Interest

The authors declare that the research was conducted in the absence of any commercial or financial relationships that could be construed as a potential conflict of interest.

## Publisher’s Note

All claims expressed in this article are solely those of the authors and do not necessarily represent those of their affiliated organizations, or those of the publisher, the editors and the reviewers. Any product that may be evaluated in this article, or claim that may be made by its manufacturer, is not guaranteed or endorsed by the publisher.

## Footnotes

^1^ http://data.tpdc.ac.cn/zh-hans/data/eac4f2cf-d527-4140-a35d-79992957f043

^2^ https://www.plantplus.cn/cn/datas/11

^3^ http://drive5.com/uparse/

^4^ http://www.genome.jp/kegg/
