# Supplementary material for: Comparative Analysis of Gut Microbial Composition and Functions in Przewalski’s Gazelle (Procapra przewalskii) From Various Habitats
Source: Front Microbiol. 2022 Jun 8;13:913358. doi: 10.3389/fmicb.2022.913358 (PMC9213746; doi:10.3389/fmicb.2022.913358)
Supplement: Supplementary file 1 [file Data_Sheet_1.PDF]

Supplementary Material

1 Supplementary Figures and Tables

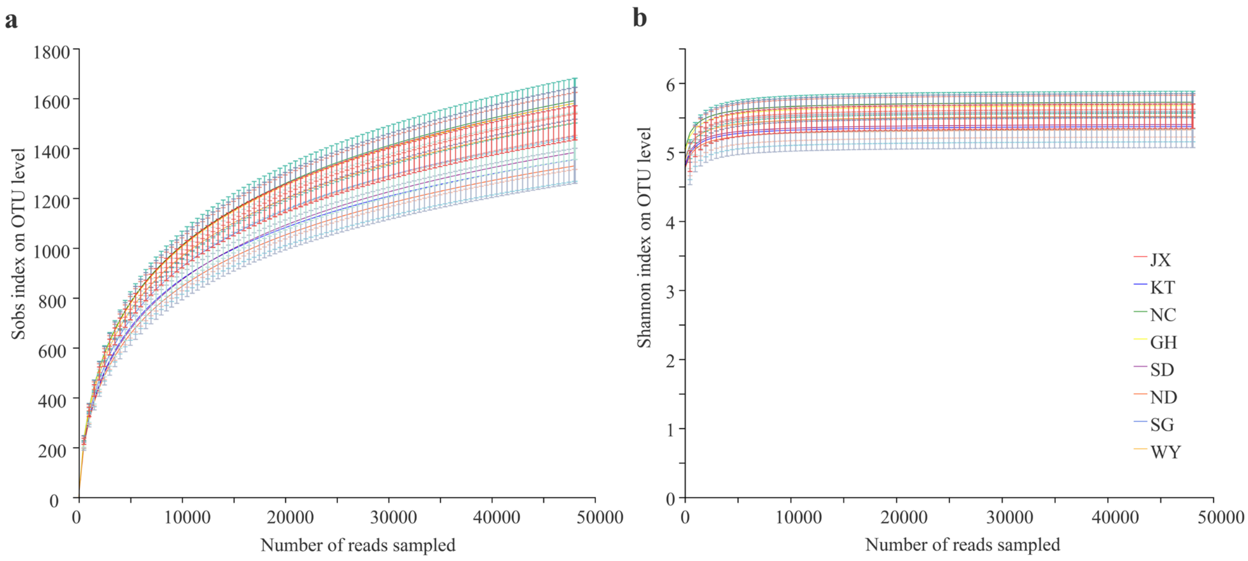

Supplementary Figure 1 Rarefaction curves of fecal sample of the Przewalski's gazelle

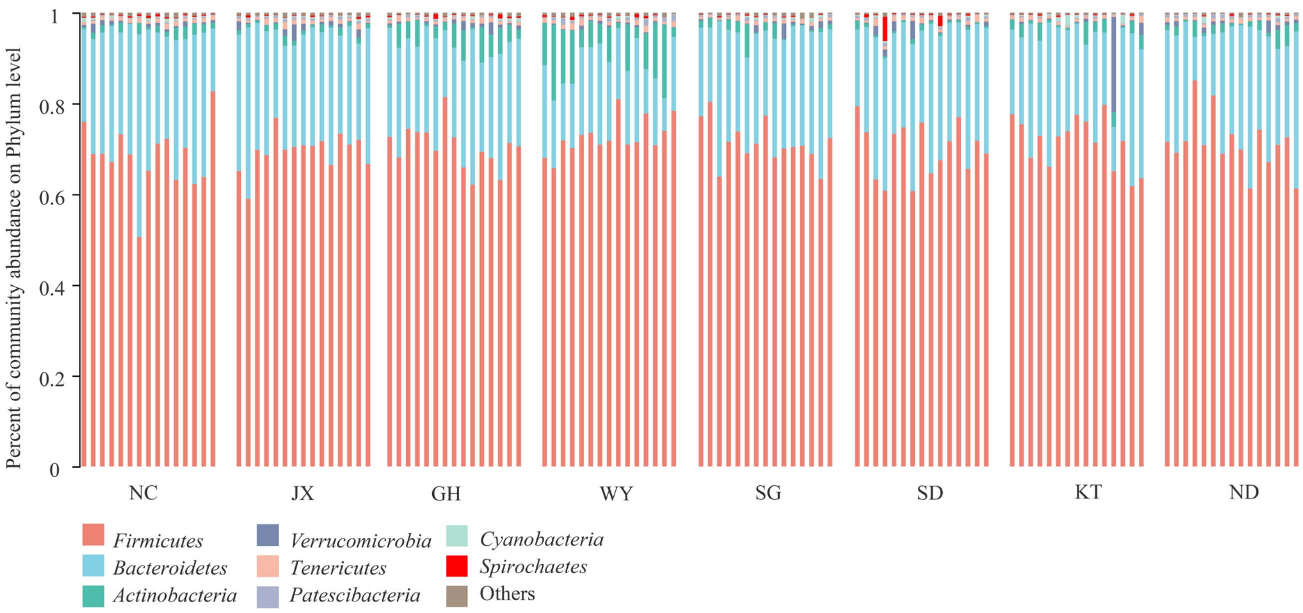

Supplementary Figure 2 Relative abundance of the dominant bacteria for each sample in each group of Przewalski’s gazelle at the phylum level.

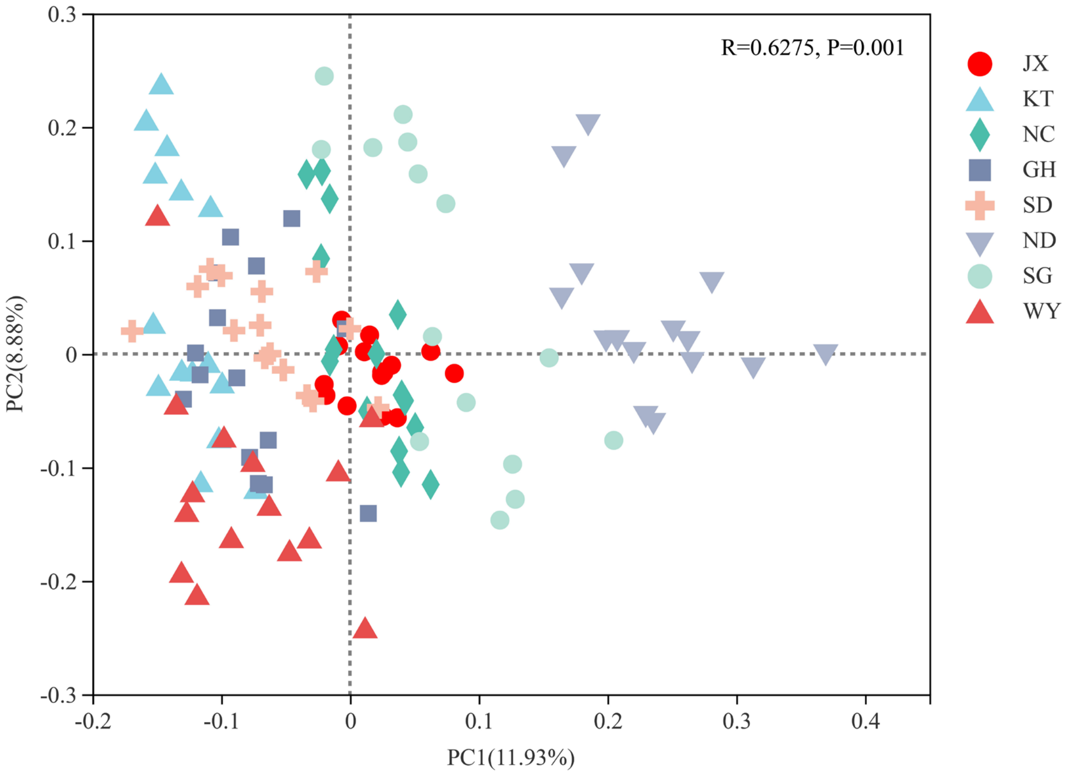

Supplementary Figure 3 PCoA analysis of gut microbiome in Przewalski's gazelle showing all samples in an ordination space using Bray Curtis.

Supplementary Table 1 PCoA analysis at the OTU level between each two groups based on three distance algorithms with Anosim and Adonnis. *: $P < 0.05$; **: $P < 0.01$; ***: $P < 0.001$.

Groups	Bray curtis		Unweighted unifrac		Weighted unifrac	
	Anosim/ R	Adonnis/ R2	Anosim/ R	Adonnis/ R2	Anosim/ R	Adonnis/ R2
JX-KT	0.84 ***	0.24 ***	0.86 ***	0.20 ***	0.40 ***	0.16 ***
JX-NC	0.53 ***	0.14 ***	0.66 ***	0.13 ***	0.14 **	0.07 *
JX-GH	0.73 ***	0.17 ***	0.75 ***	0.13 ***	0.32 ***	0.14 ***
JX-SD	0.74 ***	0.21 ***	0.90 ***	0.20 ***	0.31 ***	0.13 ***
JX-ND	0.84 ***	0.26 ***	0.96 ***	0.20 ***	0.36 ***	0.18 ***
JX-SG	0.64 ***	0.18 ***	0.81 ***	0.17 ***	0.29 ***	0.10 **
JX-WY	0.78 ***	0.22 ***	0.80 ***	0.16 ***	0.65 ***	0.34 ***
KT-NC	0.58 ***	0.18 ***	0.73 ***	0.16 ***	0.22 ***	0.12 **
KT-GH	0.53 ***	0.15 ***	0.63 ***	0.13 ***	0.18 **	0.10 **
KT-SD	0.55 ***	0.17 ***	0.52 ***	0.11 ***	0.13 ***	0.02 ***
KT-ND	0.91 ***	0.32 ***	0.91 ***	0.21 ***	0.43 ***	0.22 ***
KT-SG	0.68 ***	0.21 ***	0.74 ***	0.17 ***	0.22 ***	0.12 **
KT-WY	0.59 ***	0.20 ***	0.62 ***	0.14 ***	0.38 ***	0.22 ***
NC-GH	0.35 ***	0.11 ***	0.27 ***	0.07 ***	0.16 **	0.10 **
NC-SD	0.63 ***	0.18 ***	0.75 ***	0.15 ***	0.19 ***	0.11 **
NC-ND	0.72 ***	0.22 ***	0.86 ***	0.19 ***	0.29 ***	0.16 ***
NC-SG	0.42 ***	0.13 ***	0.47 ***	0.11 ***	0.13 *	0.07 *
NC-WY	0.58 ***	0.18 ***	0.56 ***	0.13 ***	0.55 ***	0.30 ***
GH-SD	0.63 ***	0.18 ***	0.74 ***	0.15 ***	0.29 ***	0.14 ***
GH-ND	0.88 ***	0.30 ***	0.98 ***	0.21 ***	0.44 ***	0.20 ***
GH-SG	0.58 ***	0.17 ***	0.69 ***	0.14 ***	0.22 **	0.10 *
GH-WY	0.47 ***	0.13 ***	0.54 ***	0.11 ***	0.30 ***	0.19 ***
SD-ND	0.90 ***	0.28 ***	0.92 ***	0.18 ***	0.40 ***	0.22 ***
SD-SG	0.61 ***	0.17 ***	0.67 ***	0.13 ***	0.20 **	0.11 **
SD-WY	0.67 ***	0.20 ***	0.68 ***	0.14 ***	0.56 ***	0.29 ***
ND-SG	0.64 ***	0.19 ***	0.76 ***	0.16 ***	0.28 ***	0.14 ***
ND-WY	0.90 ***	0.31 ***	0.90 ***	0.19 ***	0.62 ***	0.29 ***
SG-WY	0.65 ***	0.21 ***	0.71 ***	0.16 ***	0.49 ***	0.26 ***

Supplementary Table 2 The Kruskal-Wallis rank-sum test of each region at the level2 of KEGG pathway abundance

Pathway level1	Pathway level2	Corrected *P* value	JX-Mean (%)	NC-Mean (%)	ND-Mean (%)	KT-Mean (%)	SD-Mean (%)	SG-Mean (%)	WY-Mean (%)	GH-Mean (%)
Cellular Processes	Cell growth and death	***	1.7830	1.7740	1.7970	1.7840	1.8080	1.7920	1.6920	1.7540
Cellular Processes	Cellular community - eukaryotes	***	0.0019	0.0019	0.0020	0.0021	0.0020	0.0022	0.0022	0.0022
Cellular Processes	Transport and catabolism	***	0.3659	0.3902	0.3815	0.3487	0.3932	0.3392	0.3080	0.3389
Cellular Processes	Cell motility	*	2.6150	2.5180	2.6810	2.6640	2.5830	2.7100	2.5220	2.5830
Environmental Information Processing	Signal transduction	***	8.6260	8.5350	8.8520	8.5570	8.6430	8.8480	8.0090	8.4520
Environmental Information Processing	Membrane transport	***	9.9550	9.8140	9.9560	10.0800	9.7260	10.2000	10.5000	10.1500
Genetic Information Processing	Translation	***	5.4480	5.4550	5.3670	5.3850	5.3910	5.3610	5.2990	5.3540
Genetic Information Processing	Transcription	**	0.2260	0.2278	0.2257	0.2288	0.2253	0.2234	0.2306	0.2256
Genetic Information Processing	Replication and repair	**	5.1700	5.1730	5.2000	5.2220	5.2140	5.2030	5.1600	5.1860
Metabolism	Xenobiotics biodegradation and metabolism	***	2.8270	2.8330	2.8210	2.9480	2.7880	2.9320	3.3530	3.0850
Metabolism	Metabolism of other amino acids	***	2.1820	2.1900	2.1870	2.2130	2.1740	2.2170	2.2870	2.2390
Metabolism	Glycan biosynthesis and metabolism	***	3.1410	3.2380	3.1630	3.0300	3.2690	2.9850	2.6580	2.9170
Metabolism	Metabolism of terpenoids and polyketides	***	1.8820	1.9380	1.8210	2.0000	1.8640	1.8580	2.4530	2.1210
Metabolism	Metabolism of cofactors and vitamins	**	6.3760	6.4420	6.3300	6.3000	6.4150	6.2210	6.5040	6.3540
Organismal Systems	Sensory system	***	1.87E-07	7.48E-07	3.53E-08	6.12E-07	1.43E-07	3.22E-07	2.61E-06	1.14E-06
Organismal Systems	Environmental adaptation	***	0.2459	0.2466	0.2538	0.2441	0.2477	0.2496	0.2269	0.2398
Organismal Systems	Digestive system	***	0.2026	0.2109	0.2115	0.1902	0.2093	0.1862	0.1593	0.1832
Organismal Systems	Immune system	***	0.0683	0.0707	0.0726	0.0646	0.0679	0.0653	0.0609	0.0641

Organismal Systems	Excretory system	***	0.0263	0.0265	0.0267	0.0278	0.0275	0.0277	0.0274	0.0281
Organismal Systems	Nervous system	**	0.1468	0.1479	0.1453	0.1431	0.1462	0.1433	0.1405	0.1441
Organismal Systems	Circulatory system	*	0.0040	0.0041	0.0041	0.0042	0.0043	0.0040	0.0039	0.0041
